# Intracranial Hemorrhage Revealing Pseudohypoparathyroidism as a Cause of Fahr Syndrome

**DOI:** 10.1155/2011/407567

**Published:** 2011-11-28

**Authors:** Abhijit Swami, Giridhari Kar

**Affiliations:** Department of Medicine, Silchar Medical College, Silchar, Assam, 788014, India

## Abstract

Pseudohypoparathyroidism is an infrequently encountered disease. It is one of the causes of Fahr syndrome which also is a rare clinical entity caused by multiple diseases. A 4-year-old man hospitalized for sudden onset left hemiparesis and hypertension was diagnosed to have right thalamic and midbrain hemorrhage on plain CT scan of the head which also revealed co-existent extensive intracranial calcifications involving the basal ganglia and cerebellum bilaterally. General physical examination revealed features of Albright hereditary osteodystrophy, goitre, hypertension, left hemiparesis, and signs of cerebellar dysfunction. Laboratory findings suggested hypocalcemia, hyperphosphatemia along with high TSH, low FT_4_, low FT_3_, and high anti-TPO antibody. Though bilateral intracranial calcifications are usually encountered as an incidental radiological finding in the CT scan of brain, in this case, the patient admitted for thalamic and midbrain hemorrhage was on investigation for associated intracranial calcification, and goitre was also found to have coexisting pseudohypoparathyroidism and autoimmune hypothyroidism.

## 1. Introduction

Pseudohypoparathyroidism (PHP) is an uncommon disease which is often underdiagnosed. In 1942, Fuller Albright first introduced the term “pseudohypoparathyroidism” to describe patients who presented with parathyroid-hormone-(PTH-) resistant hypocalcaemia and hyperphosphatemia along with an unusual constellation of developmental and skeletal defects, collectively termed Albright hereditary osteodystrophy (AHO). Pseudohypoparathyroidism (PHP) and Albright's hereditary osteodystrophy (AHO) are not interchangeable terms. AHO describes a constellation of physical features, including short adult stature, obesity, brachydactyly, and ectopic ossifications. PHP means end-organ resistance to PTH and is subclassified into types Ia, Ib, and Ic and type II [1].

The incidence of PHP around the world is not known though the prevalence rate has been found to be 3.4 cases per 1 million people visiting hospitals in Japan [2].

One of the unusual findings in PHP on neuroradiological investigation is the presence of basal ganglia calcification. Fahr syndrome is a general term and was first described by Fahr in 1930. It is defined by the presence of bilateral and symmetric striatopallidal calcifications [3]. 

Though intracranial calcifications are incidentally detected on CT scan of head and are mostly benign requiring no followup, PHP is one of the causes of Fahr syndrome.

We present a case of right thalamic and midbrain hemorrhage being admitted with left hemiparesis and hypertension which on investigation for identifying the cause of Fahr syndrome was found to have PHP, AHO, and autoimmune hypothyroidism.

## 2. Case Report

Mr. JU, aged 45 years, was hospitalized with sudden-onset weakness of the left side of body and face. At the onset of his illness, he had a transient loss of consciousness and on recovery complained of generalized headache with nausea and vomited once before hospitalization. He was able to pass urine and stool voluntarily and could swallow food offered by the attendants. The patient himself was able to narrate his illness after hospitalization. He had a long history of constipation—passing hard stool every 3-4 days for which he had not taken any medications. He was a farmer, but for the last two years, he had a progressive difficulty in maintaining balance while walking for which he had stopped going to his fields. He also had diminished vision in both eyes for both near and distant objects. He never consulted a doctor for his ailments. He was married with three children, all of them had a normal height and weight for age, the eldest being 22 years old and gainfully employed. His parents were dead, and his two siblings were free of any illness and were self-employed.

**Figure 1:**
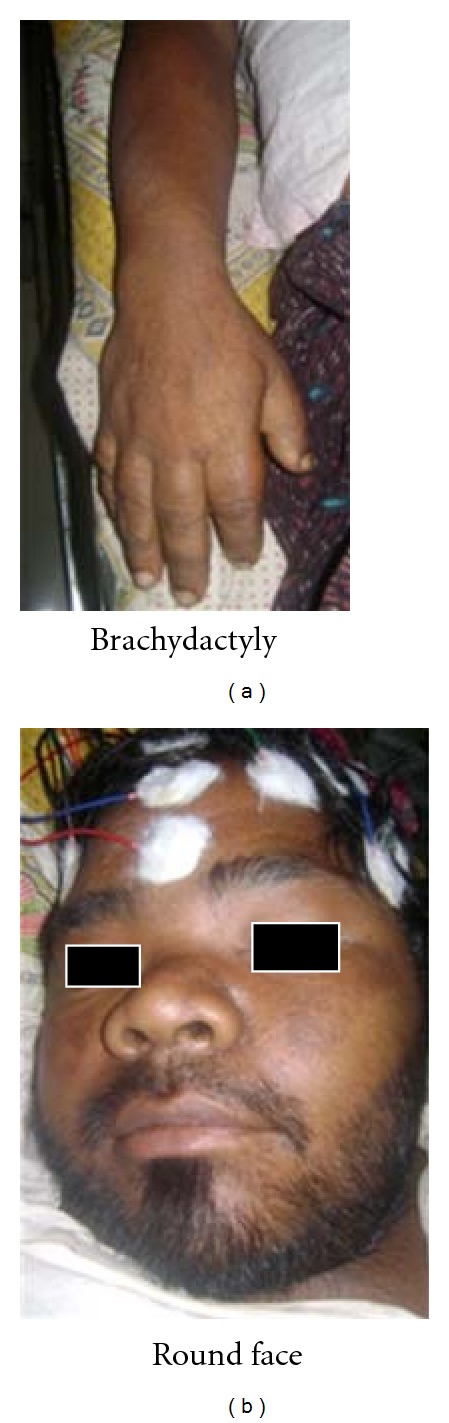


On examination, the patient had GCS 15/15 with a height of 4′10′′ and weight of 58 kg (See Figure 1). 

He had a mild degree of pallor, small goiter with coarse skin. His BP on presentation was 200/110 mmHg on both limbs with a pulse rate of 56/minute in regular rhythm with no brachiobrachial and brachiofemoral delay. He had short fingers bilaterally. His memory and orientation of time and space were normal. He had slurred speech and bidirectional nystagmus on both eyes along with left VII N and XII N upper motor lesion. Motor power was 5/5 on right side with intact sensation. He was unable to perform alternating rapid movements of the right hand and exhibited dysmetria on the right side. He had no muscle wasting and had a power of 3/5 on both upper and lower limbs muscles on the left side. On the left side, his tendon reflexes were depressed and plantar response was extensor. Right-sided patellar jerks showed normal amplitude with slow relaxation. His gait could not be tested because of the weakness of his left side. He did not have any abnormal movements. His cardiovascular system and respiratory system did not reveal any abnormality clinically. Examination of abdomen was normal. He had normal secondary sexual characteristics with normal genitalia. His musculoskeletal system examination revealed short fingers bilaterally. His spine was normal. Eye examination showed a visual acuity of 6/60 both eyes with bidirectional nystagmus. Fundus examination showed grade I hypertensive changes.

**Figure 2:**
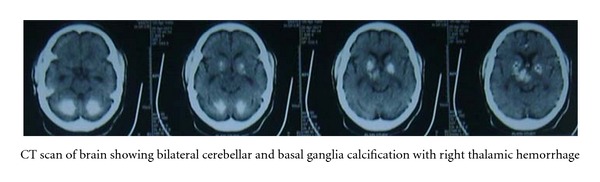
Plain CT scan of head showed bilateral basal ganglia and cerebellar calcification with right thalamic and midbrain hemorrhage without any midline shift.

Considering his presenting symptoms and examination findings, a clinical diagnosis of left hemiparesis due to cerebrovascular accident with hypertension and a possible associated hypothyroidism and cerebellar degenerative disorder was made (See Figure 2).

Hematological examination showed Hb level of 9.1 g/dL with MCV 84.9 fL, normal total and differential leucocyte count, and a normal ESR and platelet count. He had normal blood glucose, renal functions, liver enzymes, and blood electrolyte levels. He had a high LDL level of 216 mg/dL. His serum calcium and phosphorus levels were 8.2 g/dL and 4.9 mg/dL, respectively. His plasma protein was 6.8 g/dL with an albumin level of 3.9 g/dL and globulin level of 2.9 g/dL. His TSH was >150 *μ*IU/L and Free T_4 _ and Free T_3_ levels were 0.1 ng/dL and 1.8 pg/mL respectively. Se PTH (intact) was 154.5 pg/L (Range 14.0–72.0 pg/L). Anti-TPO was positive. ANA was negative. The patient tested negative for HIV. Serum iron, TIBC, and % saturation were normal.

**Figure 3:**
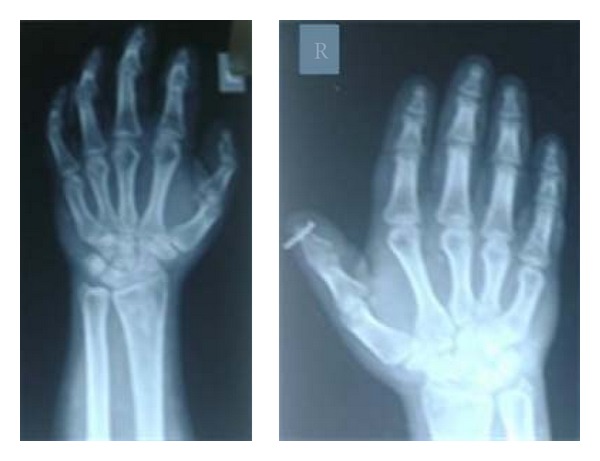
X-ray of hand showing short 3rd, 4th, and 5th metacarpals.

**Figure 4:**
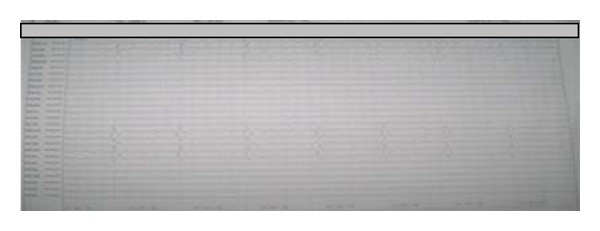
Low-voltage EEG recording with multiple ECG artefacts.

ECG showed sinus bradycardia. X-Ray hand showed short 3rd, 4^th^, and 5th metacarpals (See Figures 3 and 4). 

His PA view chest X-Ray was normal. EEG showed generalized low-voltage tracing.

The patient was treated with tab olmesartan 20 mg per day for hypertension with physiotherapy for his left-sided weakness. After his thyroid test and reports were available, the patient was given tab thyroxine 50 mcg per day initially with a plan to adjust dose at a later date after a repeat of his TSH levels at 6–8 weeks. He was also given tab atorvastatin 10 mg/day, calcium 500 mg twice daily and calcitriol 0.5 *μ*g/day. The patient improved clinically and on discharge had a muscle power of 3+/5 on his left side.

## 3. Discussion

Intracranial calcifications are encountered accidentally in 0.3–1.2% of routine radiological examinations [4]. Bilateral almost symmetric calcification involving striatum, pallidum with or without deposits in dentate nucleus, thalamus, and white matter has been reported from asymptomatic individuals as well as patients with a variety of systemic diseases and a particular neurological condition with autosomal dominant inheritance called Fahr disease [5]. Fahr syndrome is a general term and is defined by the presence of bilateral symmetrical striatopallidal calcifications at the base of the skull [3]. More than fifty reported clinical conditions have been associated with Fahr syndrome [6]. Although most of these conditions causing Fahr syndrome are systemic diseases, the reason of the focal accumulation of calcium in basal ganglia can be due to local factors and disturbance of calcium metabolism [7] 

Parathyroid diseases are one of the most common causes of Fahr syndrome. One of the commonest causes is pseudohypoparathyroidism (PHP) which is due to resistance to the action of parathyroid hormone (PTH) causing hypocalcaemia and a high level of PTH [8]. 

Pseudohypoparathyroidism (PHP) as a cause of hypoparathyroidism can cause bilateral intracranial calcifications in the basal ganglia, thalamus, cerebral white matter, and cerebellum and can be related to the neurological abnormalities like extrapyramidal signs and dyskinesia [9]. 

PHP is a hereditary disorder characterized by symptoms and signs of hypoparathyroidism, often in association with distinctive skeletal and developmental defects. PHP is classified into various forms depending on the signs of ineffective PTH action (low blood calcium and high phosphate), urinary cyclic AMP response to exogenous PTH, the presence or absence of Albright's hereditary osteodystrophy (AHO), and assays of the concentration of the G_s_ *α* subunits of the adenylate cyclase enzyme [10]. 

 AHO is a syndrome with a wide range of manifestations including short stature, obesity, round face, subcutaneous ossifications, and characteristic shortening and widening of long bones of hands and feet (brachydactyly) mostly affecting 4th and 5th metacarpals and metatarsals [11]. AHO occurs in pseudohypoparathyroidism-1a (PHP-1a). 

 Classically, patients with PHP-1a have the skeletal features of AHO, resistance to multiple hormones that work via cAMP such as PTH and TSH, and a deficient activity of Gs protein. Hypothyroidism is a manifestation of multihormonal resistance in PHP-Ia patients, and patients with PHP-Ia have impaired sensitivity to both TSH and TRH with a mild-to-moderate elevation of serum TSH concentration [12]. Hypothyroidism is generally mild and involves slightly elevated TSH concentration along with normal or slightly low thyroid hormone concentrations. The PHP-Ia patients do not have circulating antithyroid antibodies and do not develop a goiter [13]. 

The mode of inheritance of AHO has been variously described as sex-linked dominant and autosomal dominant. However, in many reported familial cases, siblings alone are affected with normal parents and autosomal recessive mode of inheritance has been postulated in such cases [14]. About half of the patients with Fahr syndrome manifest a variety of neurological deficits which includes headache, vertigo, Parkinsonism, chorea, tremor, dystonia, syncope, and seizures [15]. Purely cerebellar syndrome as a presenting feature of Fahr syndrome has also been described [16]. and there has been a case report of recurrent cerebrovascular disease along with pseudohypoparathyroidism [17]. but in the present case, the spontaneous intracranial hemorrhage was acute and could be related to coexisting hypertension and dyslipidemia. 

According to the clinical presentation, examination findings, and subsequent investigation report, our case was finally diagnosed to be a case of Fahr syndrome due to PHP-1a with associated primary autoimmune hypothyroidism and AHO, all of which were unmasked due to thalamic and midbrain hemorrhage caused by hypertension. 

We searched the literature but could not find a record of any case of PHP and Fahr syndrome with coexistent autoimmune hypothyroidism and intracerebral hemorrhage.

##  Conflict of Interests

The authers have no conflict of interests.
